# Treatment of a post-traumatic stiff knee after an open extensor apparatus injury by arthroscopic arthrolysis through a free flap

**DOI:** 10.1016/j.tcr.2021.100553

**Published:** 2021-11-11

**Authors:** Yuri Lara, Jordi Aguilera-Sáez, Jordi Tomás-Hernández, Jordi Teixidor-Serra, Andrea-Salomé Khoudeir-Ávila, José-Vicente Andrés-Peiró

**Affiliations:** a Orthopaedic Surgery Department, Hospital Universitari Vall d'Hebron, Barcelona, Spain; b Plastic Surgery Department and Burn Center, Hospital Universitari Vall d'Hebron, Barcelona, Spain; c Orthopaedic Trauma Unit, Orthopaedic Surgery Department, Hospital Universitari Vall d'Hebron, Barcelona, Spain; d Rehabilitation Medicine Department, Hospital Universitari Vall d'Hebron, Barcelona, Spain

**Keywords:** Open patella fracture, Extensor apparatus injury, Free flap, Stiff knee, Arthroscopy

## Abstract

Open patella fractures have high complication rates. Post-traumatic joint stiffness is particularly common. The management of this complication is even more difficult if free flap was used to cover a soft tissue defect. Late surgical manipulation of free flaps can lead to their failure, with catastrophic consequences. The use of minimally invasive techniques could reduce the associated risks. We present a case of knee stiffness after the fix and flap treatment of a grade IIIB open patella fracture. We performed an arthroscopic arthrolysis with portals through the flap. The pedicle was preoperatively located and avoided. Joint range of motion remarkably improved without records of flap complications. We consider that the technique is feasible. Its success was based on the multidisciplinary collaboration between orthopaedic and plastic surgeons and rehabilitation medicine specialists.

## Introduction

Open patella fractures are rare and usually occur in high-energy scenarios [1]. When a soft-tissue defect coexists, it may require a microsurgical free flap [2]. Knee stiffness is one of the most frequent complications, sometimes requiring an arthrolysis surgery [3]. However, late surgical manipulation of a successful flap can lead to its failure and a catastrophic situation [4].

We present the case of a stiff knee after the repair of an open grade IIIB patella fracture. The soft-tissue defect was covered by an Anterolateral Thigh (ALT) flap, comprising almost all the anterior aspect of the knee. The treatment consisted of an arthroscopic arthrolysis with portals through the flap.

## Case report

**Fig. 1:**
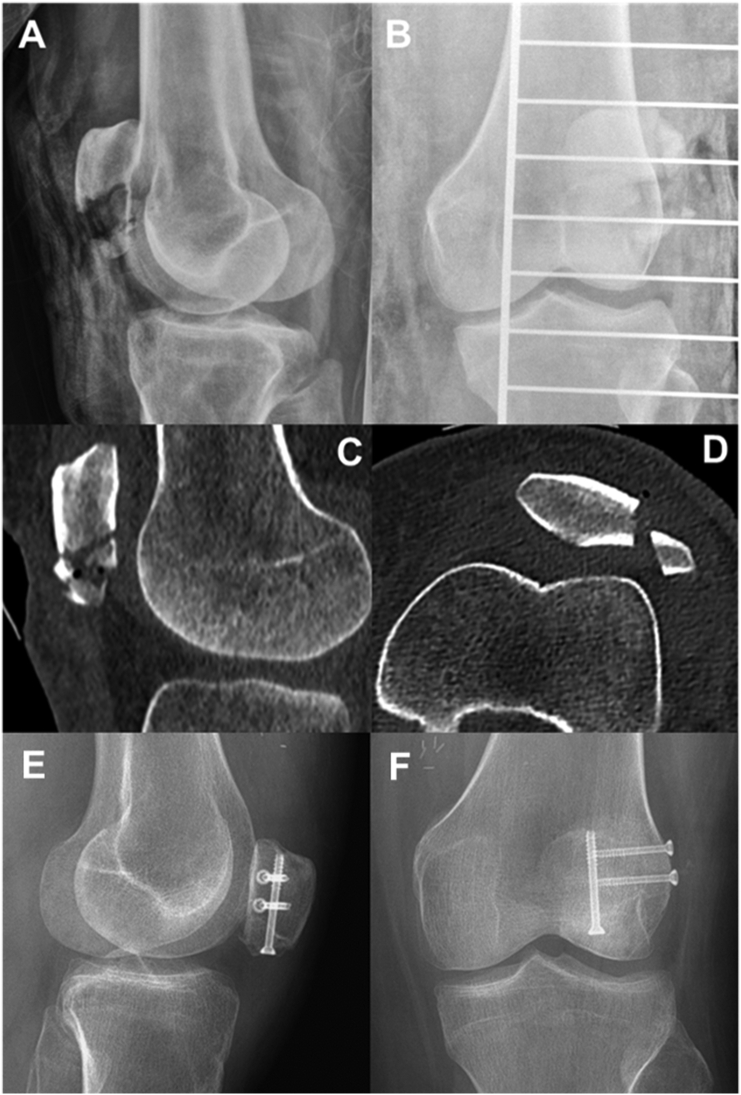
Preoperative and postoperative imaging of the original injury. A: Preoperative lateral X-ray of the knee. B: Preoperative AP X-ray of the knee. C: Preoperative sagittal CT of the knee. Important comminution is visible at the distal end of the patella. D: Preoperative axial CT of the knee. A displaced vertical fracture of the lateral facet is visible. E: Postoperative lateral X-ray of the knee at the fifth month of follow-up. Notice complete fracture healing and normal patellar height. F: Postoperative AP X-ray of the knee at the fifth month of follow-up.

**Fig. 2:**
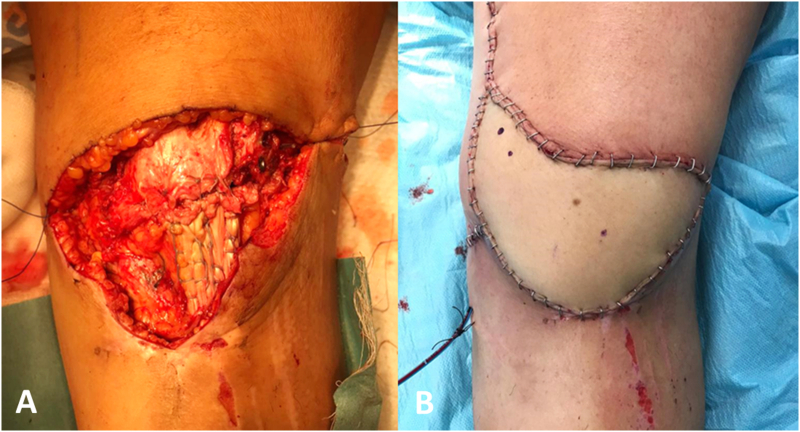
Intraoperative pictures of the orthoplastic reconstruction. A: Depiction of the soft-tissue defect and the underlying reconstruction with cannulated screws and Krakow suture. B: The area after microvascular soft-tissue reconstruction by plastic surgeons.

A 42-year-old woman suffered an open extensor apparatus injury on the left knee as the result of a motorcycle accident. The injury included a multifragmentary patella fracture (AO/OTA 34C3) and a proximal patellar tendon avulsion (Fig. 1). A 10x16cm soft-tissue defect was diagnosed after debridement. A single-step orthoplastic approach was carried out, consisting of fixation of the patella fracture with cannulated screws, transosseous reattachment of the tendon using a Krakow-type suture, and coverage with a contralateral ALT free flap (Fig. 2). The flap was vascularized by end-to-end venous and end-to-side arterial anastomosis between perforating branches and the distal superficial femoral vessels. It needed anastomotic revision during the first 24 h due to pedicle thrombosis. After two weeks of rest and limb elevation, weight-bearing was initiated. Hospital discharge was achieved after 20 days without complications. Low-molecular-weight heparins were used for deep venous thrombosis prophylaxis for 6 weeks.

The limb was immobilized in extension for 6 weeks. Then, intensive physical therapy began. Despite complete adherence, the patient developed a stiffness of the knee (flexion 70°) five months after surgery. Radiographs showed a consolidated fracture and correct patellar height, and the flap was in perfect condition. The case was discussed with plastic surgeons. The decision was to perform an arthroscopic arthrolysis with portals through the flap itself after pedicle localization with vascular Doppler.

**Fig. 3:**
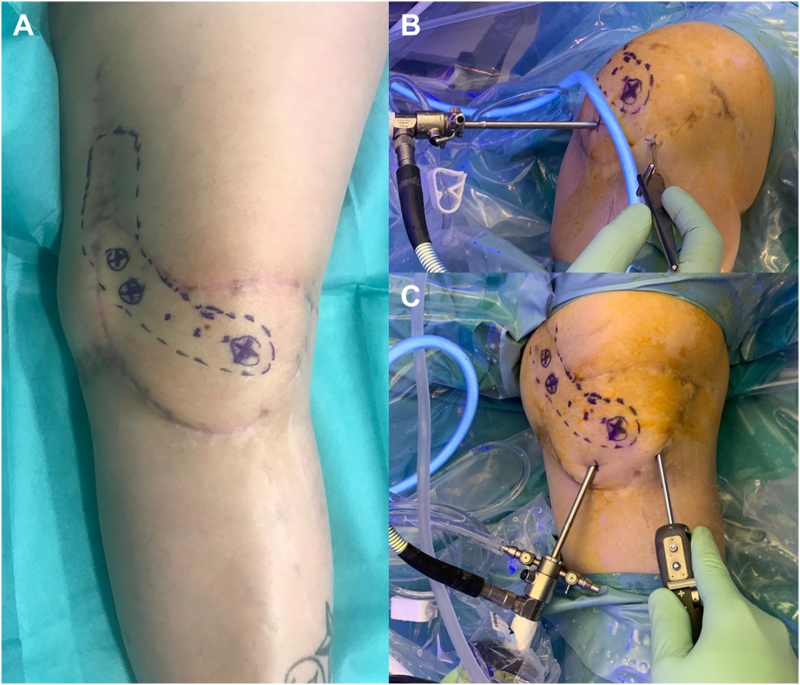
Intraoperative pictures of the arthroscopic arthrolysis. A: Marked trajectory of the free flap pedicle. B: The medial parapatellar portal passes through the free flap. C: Knee extension is necessary to work on the suprapatellar area and to perform the bilateral retinacular release.

**Fig. 4:**
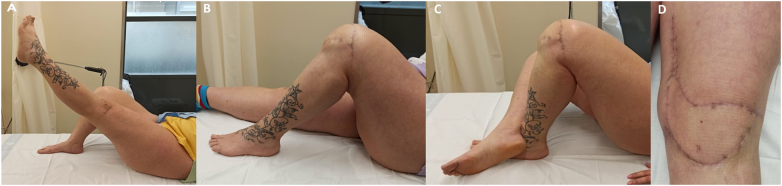
Clinical pictures at the end of follow-up. A: Knee active full extension without extensor lag. B: Knee active full flexion. C: Knee forced full flexion. D: The flap looks vital without signs of venous congestion. The arthroscopic portals are completely healed.

Once in the theater, two standard medial and lateral infrapatellar arthroscopic portals were made. The medial portal passed through the flap in an area away from the pedicle (Fig. 3). Inside the joint, significant fibrosis was found. A wide synovectomy was performed and both retinacula were sectioned under direct vision. Complete release was noticeable due to the great improvement in patellar translation and in range of motion to 90°. The flap showed correct signs of vitality after tourniquet release. Knee flexion was forced, achieving a full range of motion effortlessly. A drainage was used for the first two postoperative days. The rehabilitation began at 24 h, including intensive physiotherapy, manipulation, and the use of a continuous passive movement (CPM) device. The patient remained hospitalized for 15 days, not experiencing complications. The endpoint active joint balance was 0 to 100°, reaching 120° of forced flexion (Fig. 4). The patient returned to her previous activities and was fully satisfied with the result.

## Discussion

9% of patella fractures present as open injuries, usually in high-energy contexts and being highly comminuted. 7.5% of them associate a soft-tissue defect (Gustilo IIIB). Open patella fractures have a higher incidence of complications compared to closed, standing out infection, non-union, joint stiffness and extensor lag. Up to 65% require additional surgeries, the most frequent being soft-tissue reconstruction procedures [1], [5].

Knee stiffness is the most frequent complication after patella fractures. It is highly associated with open fractures, infections, or inadequate rehabilitation protocols with prolonged immobilization [6], [7]. The greater severity of the soft-tissue injury in open fractures facilitate arthrofibrosis and extra-articular adhesions (subcutaneous and quadriceps) [3]. When conservative treatment fails, arthroscopic arthrolysis is the treatment of choice because of its minimally invasiveness. The outcomes are better if done during the first 6 months of follow-up [3], [7]. Relative contraindications to this procedure are unresolved injuries to the extensor apparatus (such as an unconsolidated patella fracture), flexion contracture of the knee, and prior failure of arthroscopic treatment [3], [6], [7]. Complications usually occur in relation to the forced movement done at the end of the procedure (fractures around the knee, cartilage injuries, and extensor apparatus disruption). An intensive physical therapy program after the procedure is mandatory [6].

The reconstruction of soft-tissue defects on the proximal pole of the patella or quadriceps tendon is not possible with conventional rotational flaps. The alteration of vascularization by the high energy trauma also discourages the use of local or regional flaps on fractures. Considering this, a fix and flap approach with skeletal stabilization and immediate free flap coverage is the best treatment for open Gustilo IIIB fractures of the patella [2]. The ALT free flap is the most used free flap to cover soft-tissue defects in the lower extremity with a success rate over 90%. It is a versatile technique which can be harvested as a fasciocutaneous or myocutaneos flap or be configured as a chimeric flap. The pedicle may be easily harvested as a flow-through [2]. The time in which a flap acquires a vascularization independent of its pedicle depends on the characteristics of the receiving area, the flap itself (type and volume), the patient (comorbidities or smoking) and external agents (infection or radiation). It may take 4 to 12 weeks for the flap to be autonomous, although it may never become so. Therefore, secondary surgical manipulation of a successful free flap can cause complications and failure [4]. It will always be advisable to avoid the pedicle area and the manipulation should only be done in strictly necessary situations [8], [2]. As a result of this, in recent years several minimally invasive techniques have been successfully applied for secondary contouring of flaps. Liposuction or the use of arthroscopic shavers may reduce the risk of failure relative to reintervention [9], [10].

Our patient developed an articular knee stiffness in a time frame in which we may consider an arthroscopic procedure (before 6 months). The extensor apparatus was healed, and the flap showed correct signs of vitality. We considered that the risk-benefit of an open procedure was not favorable. However, the experiences reported on the use of minimally invasive techniques in flap debulking led us to consider practicing arthroscopic portals through the flap itself. The procedure was carried out as described and we obtained a satisfactory clinical result.

## Conclusion

Arthroscopic arthrolysis of the knee through a free flap is a feasible technique when vascular pedicle is avoided. The success of this case, both the initial reconstruction and the release surgery, must be attributed to the multidisciplinary collaboration between orthopaedic and plastic surgeons and the rehabilitation team.

## Consent

Patient's consent was obtained for the elaboration of this work. This case report was conducted in accordance with the Declaration of Helsinki on ethical principles for medical research.

## Funding

There was no funding for the preparation of this work.

## Declaration of competing interest

The authors declare that there is no competing conflict of interest.
